# Insight of the Interaction between 2,4-thiazolidinedione and Human Serum Albumin: A Spectroscopic, Thermodynamic and Molecular Docking Study

**DOI:** 10.3390/ijms20112727

**Published:** 2019-06-03

**Authors:** Safikur Rahman, Md Tabish Rehman, Gulam Rabbani, Parvez Khan, Mohamed F AlAjmi, Md. Imtaiyaz Hassan, Ghazala Muteeb, Jihoe Kim

**Affiliations:** 1 Department of Medical Biotechnology, Yeungnam University, Gyeongsan 712-749, Korea; shafique2@gmail.com; 2 Department of Pharmacognosy, College of Pharmacy, King Saud University, Riyadh 11451, Saudi Arabia; mrehman@ksu.edu.sa (M.T.R.); malajmii@ksu.edu.sa (M.F.A.); 3 Nano Diagnostics; Devices (NDD), Room B-312 IT, Medical Fusion Center, Gumidae-ro, 350-27, Gumi-si, Gyeongbuk 39253, Korea; rbbgulam@gmail.com; 4 Center for Interdisciplinary Research in Basic Sciences, Jamia Millia Islamia, Jamia Nagar, New Delhi 110025, India; parvezynr@gmail.com (P.K.); mihassan@jmi.ac.in (I.H.); 5 Department of Nursing, College of Applied Medical Sciences, King Faisal University, 31982 Al-Ahsa, Saudi Arabia

**Keywords:** Thiazolidinedione, human serum albumin, antidiabetic, thermodynamic stability

## Abstract

Thiazolidinedione derivatives (TZDs) have attracted attention because of their pharmacological effects. For example, certain TZDs have been reported to ameliorate type II diabetes by binding and activating PPARs (peroxisome proliferator-activated receptors). Nonetheless, no information is available on the interaction between the heterocyclic 2, 4-thiazolidinedione (2,4-TZD) moiety and serum albumin, which could affect the pharmacokinetics and pharmacodynamics of TZDs. In this study, we investigated the binding of 2,4-TZD to human serum albumin (HSA). Intrinsic fluorescence spectroscopy revealed a 1:1 binding stoichiometry between 2,4-TZD and HSA with a binding constant (*K_b_*) of 1.69 ± 0.15 × 10^3^ M^−1^ at 298 K. Isothermal titration calorimetry studies showed that 2,4-TZD/HSA binding was an exothermic and spontaneous reaction. Molecular docking analysis revealed that 2,4-TZD binds to HSA subdomain IB and that the complex formed is stabilized by van der Waal’s interactions and hydrogen bonds. Molecular dynamics simulation confirmed the stability of the HSA-TZD complex. Further, circular dichroism and 3D fluorescence studies showed that the global conformation of HSA was slightly altered by 2,4-TZD binding, enhancing its stability. The results obtained herein further help in understanding the pharmacokinetic properties of thiazolidinedione.

## 1. Introduction

Thiazolidinedione derivatives (TZDs) are oral hypoglycemic compounds that are commonly used alone or in combination with other drugs to manage type 2 diabetes mellitus. Representative drugs that contain the 2,4-thiazolidinedione (2,4-TZD) moiety include pioglitazone, rosiglitazone, and troglitazone, which bind to and activate PPAR-γ, which in turn, regulates adipocyte differentiation and carbohydrate and fatty acid metabolism [1,2,3,4,5,6]. TZDs have also been reported to be linked with increased risks of cancer and cardiovascular diseases, for example, pioglitazone has been shown to promote bladder cancer in patients suffering from type 2 diabetes mellitus [3]. Nevertheless, not all TZDs have been associated with cancer risk, and some TZDs have been shown to inhibit proliferation of various cancers [7]. TZDs have also been reported to modulate the activity of IKK-β kinase, and hence, the NF-κB-signaling pathway, which controls the expressions of many genes involved in inflammation, oncogenesis, apoptosis, viral infections, and immune response [8].

Human serum albumin (HSA) is the most abundant serum protein (constitutes 60% of total serum protein) which binds and transports many exogenous and endogenous molecules. HSA is a single chain polypeptide of 585 amino acid residues and has a heart-shaped three-dimensional conformation [9,10]. Its structure can be divided into three similar alpha helical subunits (domains I, II, and III) and each of these domains is composed of two sub-domains (represented as A, and B) [11]. The three domains of HSA are bound by 17 disulfide bridges [12]. HSA contains two principal ligand-binding cavities, that is, Sudlow’s site I and II, which are located in subdomains IIA and IIIA, respectively. In addition, another ligand binding site was identified and located in subdomain IB [13]. The opening of site I is surrounded by basic residues (Lys195, Lys199, Arg218, Arg222); however, the bottom is hydrophobic in nature. Additionally, Trp214, an important residue for structural analysis is also located in site I. The comparatively small site II predominantly has hydrophobic residues and accommodates the binding of hydrophobic drugs. HSA has been shown to bind various drugs including TZDs [14,15,16,17,18]. The crystal structure revealed the binding of endogenous (e.g., heme, bilirubin etc.) as well as exogenous (phenylbutazone, warfarin etc.) ligands to HSA in site I and II [19,20,21,22]. Recently, we also showed that HSA binds with cyclobenzaprine hydrochloride [23], eperisone hydrochloride [15], erucic acid [24], and tolperisone hydrochloride [25] by using various biophysical and *in-silico* techniques.

The binding of ligands to serum protein modulates their pharmacodynamics and pharmacokinetics. Since pharmacokinetic properties such as rate of uptake and clearance of drug depends on how it interacts with HSA, it becomes vital to explore the binding affinity of the drug [26,27]. As the uptake of the drug occurs in an unbound form, the pharmacodynamic property of drug is controlled by the balance of the active concentration of the drug to its reversible binding to HSA [28]. Several HSA–ligand binding experiments revealed the binding affinity (binding constant (*K_b_*)) in the range of ~10^2^ to 10^6^ M^−1^ [9,29,30,31,32,33,34,35,36]. For instance, HSA binds pioglitazone with high affinity (*K_b_* = 1.1 × 10^5^ M^−1^) [33]. However, moderate affinity (*K_b_* = 6.25 × 10^2^ M^−1^) was determined for the binding of rosiglitazone to a HSA homolog, bovine serum albumin [36]. Thus, it is important that the interaction between 2,4-TZD and HSA is understood.

In this study, we employed spectroscopic, thermodynamic, and molecular docking approaches to identify the mechanism by which 2,4-TZD binds with HSA. We found that 2,4-TZD binds with the IB site of HSA and that binding alters the conformation and thermodynamic stability of HSA. These findings advance the understanding of the interaction between 2,4-TZD and HSA.

## 2. Results and Discussion

### 2.1. Characterization of 2,4-TZD Binding Sites on HSA

#### 2.1.1. Mechanism of HSA Fluorescence Quenching by 2,4-TZD

**Figure 1:**
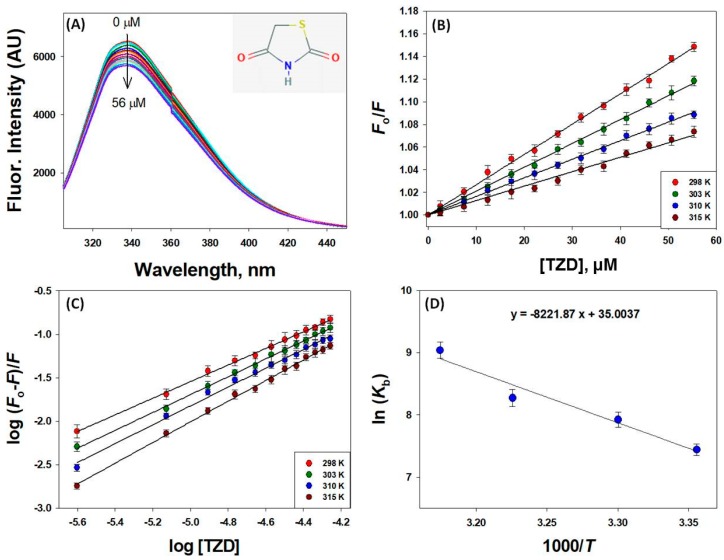
Intrinsic fluorescence of HSA in the presence of 2,4-TZD at different temperatures. (**A**) Fluorescence quenching as a function of 2,4-TZD concentration at 298 K. The inset shows the structure of 2,4-TZD, (**B**) Stern-Volmer plot of HSA quenching by 2,4-TZD, (**C**) Double logarithm or modified Stern-Volmer plot, and (**D**) a van’t Hoff plot, which was used to calculate thermodynamic parameters. *n* = 3.

To investigate the 2,4-TZD to HSA binding, fluorescence emission quenching experiments were conducted in the presence or absence of 2,4-TZD at pH 7.4 at four different temperatures (298, 303, 310, and 315 K). Concentration-dependent decreases in the fluorescence intensity of HSA were observed (emission maxima at 340 nm) upon adding 2,4-TZD at concentrations of 0 to 56 µM at 298 K (Figure 1A). Similarly, HSA fluorescence showed similar decreases in fluorescence intensities at other temperatures (303, 310, and 315 K), which suggested 2,4-TZD bound at a site close to Trp214 (Tryptophan 214). A detail of the binding mechanism was also obtained using the Stern-Volmer equation:

(1) $$\frac{F_{o}}{F}=1+K_{SV}\left[Q\right]=1+k_{q}\tau_{o}\left[Q\right]$$

where *F_o_* is the experimentally observed fluorescence intensity of free HSA, *F* is the fluorescence intensity observed during 2,4-TZD titration, *k_q_* is the bimolecular quenching rate constant, *τ_o_* is the average fluorescence lifetime of HSA in the absence of quencher, *K_SV_* is the Stern-Volmer quenching constant, and [*Q*] is the molar concentration of quencher.

Binding study results showed that the *K_SV_* (Stern-Volmer constant) of 2,4-TZD for HSA at all studied temperatures was of the order 10^3^ M^−1^ (Figure 1B, Table 1), indicating moderate interaction between HSA and 2,4-TZD. Linear regression analysis of Stern-Volmer plots of *F_o_*/*F* against the concentration of 2,4-TZD [2,4-TZD] at four different temperatures showed an inverse relation with temperature and *K*sv value. This finding indicated that quenching of HSA was static rather than dynamic and initiated by 2,4-TZD-HSA complex formation (Figure 1B).

The temperature-dependent bimolecular quenching rate constant (*k_q_*) was calculated by dividing *K_SV_* by *τ_o_*, which was taken to be 5.71 × 10^−9^ s for HSA, as previously reported [37]. The *k_q_* value of the HSA-2,4-TZD complex was of the order of 10^11^ M^−1^ s^−1^, i.e., ≥ 10 times the diffusion constant (2 × 10^10^ M^−1^ s^−1^), suggesting that quenching of HSA by 2,4-TZD was due to the formation of a ground state complex. The observed dependency of the quenching process on temperature supported this suggestion, because it has been well established that dynamic quenching *K*sv values increase with temperature due to higher kinetic energy, whereas static quenching reduces *K*sv values at higher temperature by inducing complex dissociation. At elevated temperatures (303, 310, and 315 K), both *K_SV_* and *k_q_* were decreased, which was ascribed to 2,4-TZD-HSA complex breakdown. These observations showed that the observed HSA fluorescence quenching was static in nature (Table 1).

#### 2.1.2. Binding and Thermodynamics of 2,4-TZD/HSA Binding

The binding constant (*K_b_*) and binding stoichiometry (*n*) of 2,4-TZD/HSA binding for static quenching were determined from the intercept and slope, respectively, of the following modified Stern-Volmer equation,

(2) $$log\left(\frac{F_{o}-F}{F}\right)=logK_{b}+nlog\left[Q\right]$$

The binding stoichiometry (*n*) of 2,4-TZD/HSA binding was close to 1 at all temperatures studied. Conversely, the binding constant (*K_b_*) varied within 1.69–8.42 × 10^3^ M^−1^ at different temperatures, indicating moderate binding between 2,4-TZD and HSA (Figure 1C and Table 1). Binding constants for various ligand/HSA interactions have been reported to vary between 10^2^–10^6^ M^−1^ [32,38,39,40]. In addition, we evaluated the thermodynamics of 2,4-TZD/HSA binding by monitoring fluorescence quenching at different temperatures, which helped us determine the natures of forces responsible for complex formation. To elucidate the binding energies involved, thermodynamic parameters (Δ*H^o^*, *T*Δ*S^o^*, and Δ*G^o^*) were obtained using van’t Hoff equations,

(3) $$\mathrm{In}K_{b}=\left(\frac{-\Delta H^{o}}{R}\right)\frac{1}{T}+\frac{\Delta S^{o}}{R}$$

(4) $$\Delta G^{o}=\Delta H^{o}-T\Delta S^{o}$$

A van’t Hoff plot of ln *K_b_* versus 1/*T* is shown in Figure 1D. Linear analysis of the van’t Hoff plot provided values for −Δ*H^o^*/*R* and Δ*S^o^*/*R* and estimated values for Δ*H^o^* and *T*Δ*S^o^* at 298 *K* of 16.34 ± 0.96 kcal mol^−1^ and 20.73 ± 1.32 kcal mol^−1^, respectively, and an estimated Δ*G^o^* of −4.39 ± 0.63 kcal mol^−1^.

#### 2.1.3. Isothermal Titration Calorimetry (ITC) Measurements

**Figure 2:**
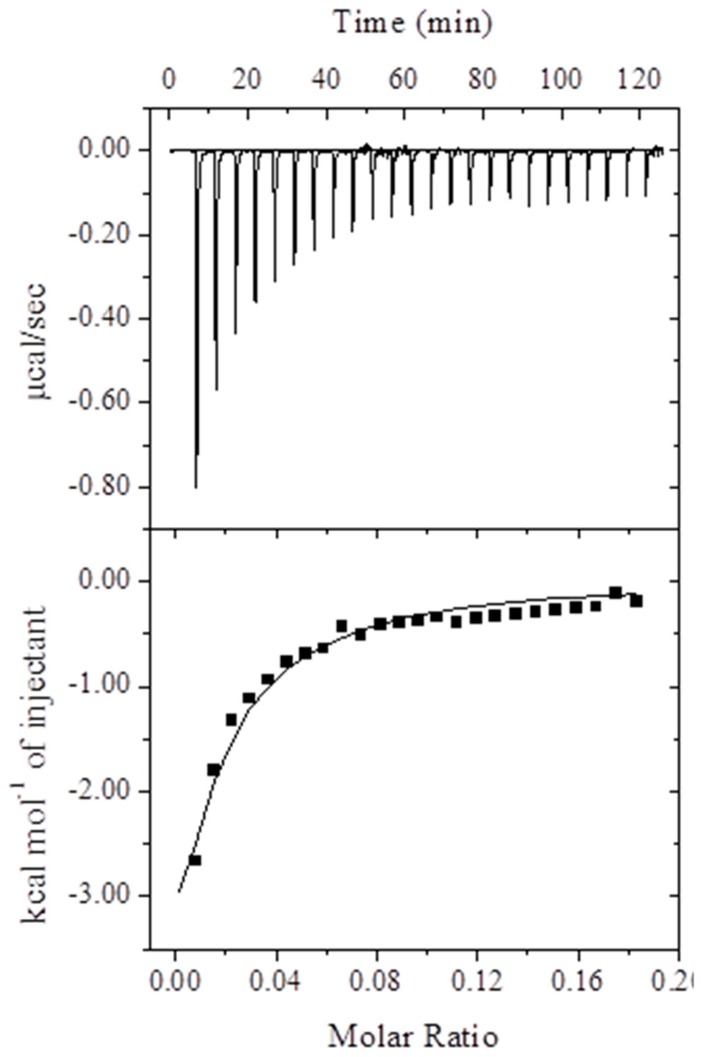
ITC isotherm of 2,4-TZD/HSA binding. The upper panel shows heat changes that took place during each injection. The lower panel demonstrates the changes in energy that took place per injection as a function of 2,4-TZD to HSA molar ratio.

The binding affinity, stoichiometry, and energetics of 2,4-TZD to HSA binding were determined by ITC. Analysis of the data in Figure 2 using an ‘One-site’ binding model revealed the binding was energetically favorable. Thermodynamic parameters determined by analyzing the data in Figure 2 are summarized in Table 2. The negative values of enthalpy (Δ*H^o^*) and entropy changes (*T*Δ*S^o^*) suggest the involvement of both hydrogen bonds as well as van der Waal’s interaction in stabilizing the HSA-2,4-TZD complex. The formation of the 2,4-TZD-HSA complex was spontaneous as suggested by a negative Gibb’s free energy change (Δ*G^o^*), and the whole binding process resulted in an energy release i.e., exothermic process.

ITC results differed considerably from the results obtained by fluorescence spectroscopy possibly due to the different approaches used. Binding and thermodynamic parameters were obtained by fluorescence spectroscopy due to the energy transfer between HSA (Trp214) and 2,4-TZD [41]. ITC provides direct measures of global phenomenon wherein energy is released/consumed as a result of an interaction between a ligand and its receptor. Further, the major drawback of non-calorimetric techniques, such as fluorescence spectroscopy, with respect to the calculation of binding and thermodynamic parameters, is that an assumption is made that entropy does not change significantly over the studied temperature range [35]. However, fluorescence spectroscopy has been widely used to measure quenching at different temperatures to obtain preliminary estimations of binding energy and thermodynamic parameters.

#### 2.1.4. Förster Resonance Energy Transfer (FRET) between HSA and 2,4-TZD

FRET is the method of choice used to measure the distance between a donor and an acceptor molecule when the fluorescence spectrum of the donor molecule overlaps with the absorption spectrum of the acceptor molecule [42]. The distance (*r*) between Trp214 of HSA (donor) and 2,4-TZD (acceptor) in HSA can be calculated using Förster’s theory [43]. The efficiency of energy transfer (*E*) is related to the critical distance or Förster radius (*R_o_*), which can be calculated using the following equation:

(5) $$E=\frac{R_{o}^{6}}{R_{o}^{6}+r_{o}^{6}}=1-\frac{F}{F_{o}}$$

where, *F_o_* and *F* are the experimentally observed fluorescence intensities of the donor (free HSA) and acceptor (HSA-2,4-TZD), *r* is the distance between donor and acceptor molecules, and *R_o_* is the critical distance between donor and acceptor molecules at which the energy transfer efficiency is 50%.

*R_o_* is calculated using the following equation.

(6) $$R_{o}^{6}=8.79\times 10^{-25}K^{2}n^{-4}\Phi J$$

where, *K*^2^ defines the geometry of donor and acceptor dipoles, *n* is the refractive index of the medium, Φ is the fluorescence quantum yield of the donor, and *J* is the area under the fluorescence spectral overlap between the donor emission and acceptor absorption. *J* is defined as

(7) $$J=\frac{\int_{0}^{\infty }F_{\lambda }\varepsilon_{\lambda }.\lambda^{4}d\lambda }{\int_{0}^{\infty }F_{\lambda }.d\lambda }$$

where, *F_λ_* is the fluorescence intensity of the donor at wavelengths of *λ* to *λ* + Δ*λ*, and *ε_λ_* is the molar absorption coefficient of the acceptor at wavelength *λ*.

In the present study for HSA, the values of *K*^2^, Φ, and *n* were taken from a previous study as 2/3, 0.118, and 1.33, respectively [44]. The values of *J*, *R_o_* and *r* were estimated to be 3.08 × 10^−14^ M^−1^ cm^3^, 2.96 nm, and 4.08, nm respectively (Table 3). It is interesting to note that the values of *R_o_* and *r* were on the 2–8 nm scale. Furthermore, validation of the relation 0.5*R_o_* < *r* < 1.5*R_o_* indicated static quenching of HSA by 2,4-TZD [42].

#### 2.1.5. Location of the 2,4-TZD Binding Site on HSA by Molecular Docking

**Figure 3:**
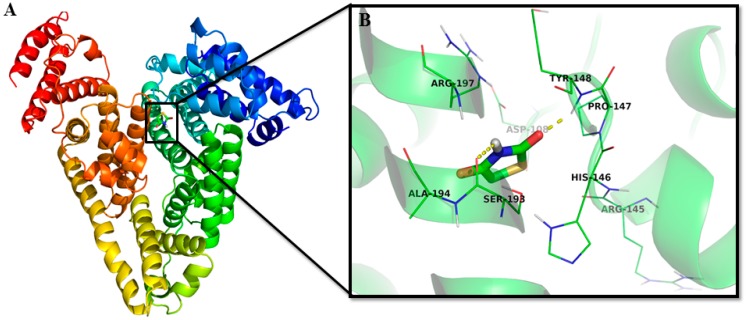
Molecular interactions between 2,4-TZD and HSA. (**A**) The binding site of 2,4-TZD at subdomain IB of HSA, and (**B**) Amino acid residues and the nature of the interaction between 2,4-TZD and HSA.

Molecular docking provides a powerful means of identifying ligand-binding sites in proteins and of investigating molecular interactions that stabilize protein–ligand complexes. Molecular docking was performed using AutoDock4.2 [45], and results are presented in Figure 3 and Table 4. Docking studies indicated that 2,4-TZD binds at the subdomain IB of HSA near Trp214 (Figure 3). Two conventional hydrogen bonds at Tyr148 (2.07 Å) and Ser193 (2.57 Å) and a carbon–hydrogen bond at Ala194 (3.38 Å) stabilized the 2,4-TZD-HSA complex. Asp108, Arg145, His146, Pro147, and Arg197 were also found to be involved in the interaction. The binding-free energy of 2,4-TZD-HSA complex formation was −4.61 ± 0.54 kcal mol^−1^, which corresponded to a binding affinity of 1.69 ± 0.15× 10^3^ M^−1^ (Table 4). These results agree well with the results obtained from fluorescence quenching experiments. In previous studies, interactions between pioglitazone and rosiglitazone with serum albumins showed that H bonds were involved in the formation of HSA–drug complexes. Additionally, rosiglitazone was found to bind to bovine serum albumin at Sudlow’s site I, whereas pioglitazone bound with HSA at subdomains IIA and IIIA [33,36].

### 2.2. Effect of 2,4-TZD on the Conformation of HSA

In general, binding of a ligand to protein induces conformational changes that can easily be monitored by circular dichroism (CD), synchronous and 3D fluorescence spectroscopy.

#### 2.2.1. Far-UV Circular Dichroism (CD) Analysis

**Figure 4:**
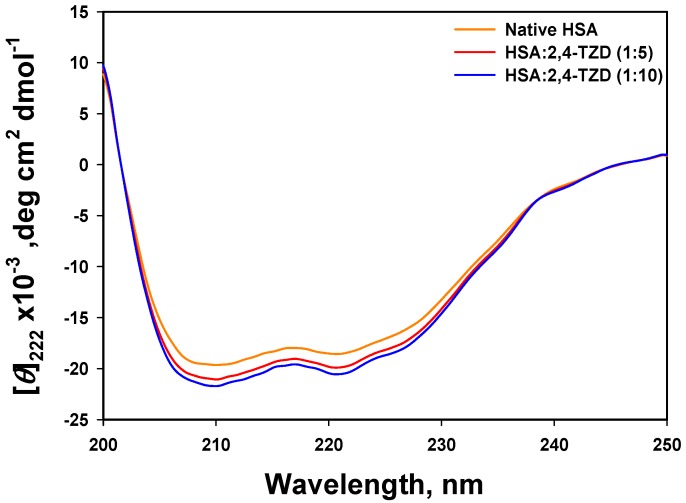
Far-UV Circular dichroism spectra of HSA at different HSA to 2,4-TZD molar ratios.

Far UV CD is widely used to determine protein conformation at the secondary structure level. To investigate 2,4-TZD-induced changes in the structure of HSA, far-UV CD was performed in the presence or absence of 2,4-TZD at pH 7.4 and 25 °C. Figure 4 shows the far-UV CD spectra of the free HSA and 2,4-TZD-HSA complex at molar ratios of 1:5 and 1:10. Each CD spectrum was analyzed for secondary structural content using the Chen method [46]. Far-UV CD of free HSA exhibited double minima at 208 nm (π →π transition) and 222 (n→π transition), which are characteristics of an α-helix rich protein. It should be noted that the α-helix content reported here was lower than that reported in the x-ray crystal structure of HSA (Pdb Id-1AO6), but agreed well with earlier published results [34,47]. This disagreement was ascribed to instrumental differences and by different structural arrangements in the solid (X-ray diffraction) and aqueous state (CD spectroscopy). On adding 20 and 40 μM of 2,4-TZD to 4 μM HSA, α-helical content increased (i.e., 53% in free HSA and 56 and 58 % in the presence of 20 and 40 μM of 2,4-TZD, respectively), which suggested a conformational change in HSA due to complex formation.

#### 2.2.2. Synchronous Fluorescence Analysis

**Figure 5:**
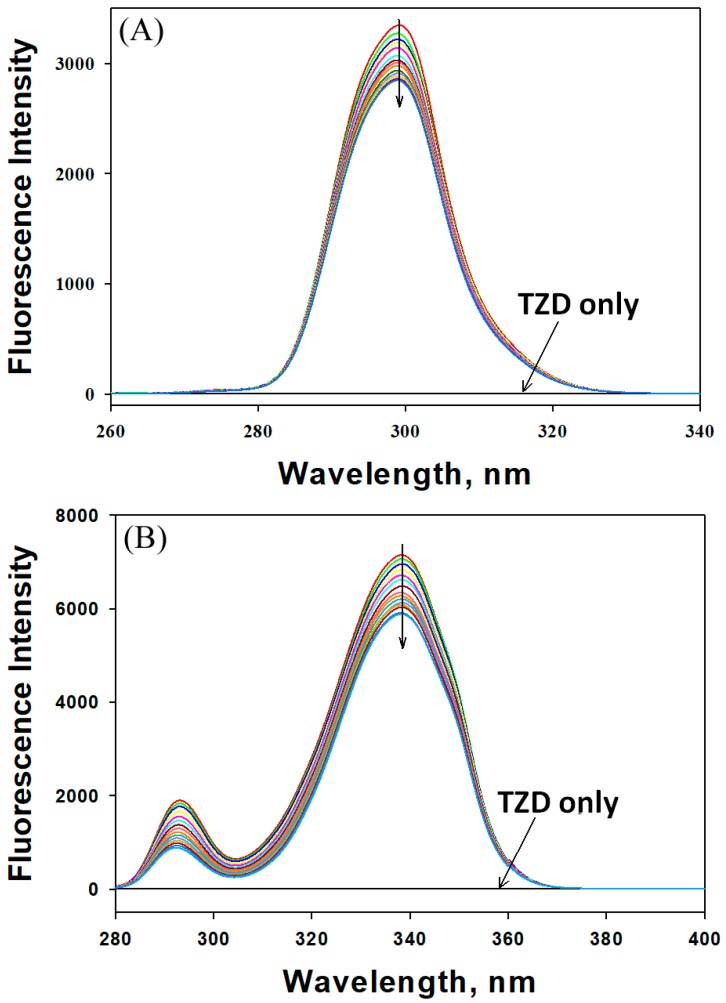
Synchronous fluorescence spectra of HSA in the absence or presence of different concentrations of 2,4-TZD. The difference in excitation and emission wavelength (Δλ) was (**A**) 15 nm for the Tyr microenvironment and (**B**) 60 nm for the Trp microenvironment.

Synchronous fluorescence spectroscopy is used to evaluate changes in protein conformation in the vicinities of aromatic amino acid residues, particularly Trp and Tyr. It is recorded by simultaneously scanning the excitation and emission monochromators at a particular fixed wavelength. When the difference between excitation and emission wavelengths, i.e., Δλ, was fixed at 60 or 15 nm, the synchronous fluorescence spectra provided information about the microenvironment around Trp and Tyr residues, respectively. Figure 5 shows the synchronous fluorescence of HSA in the absence or presence of different concentrations of 2,4-TZD. When Δλ was set to 15 nm, the fluorescence intensity of HSA in the absence of 2,4-TZD (3348 au at 299.2 nm) was quenched by 14.96% to 2847 au and blue shifted by 0.4 nm in the presence of 56 µM 2,4-TZD. Similarly, when Δλ was set to 60 nm, the fluorescence intensity of HSA in the absence of 2,4-TZD (7150 au at 338.2 nm) was quenched by 17.65% to 5888 au with no apparent shift in the wavelength maxima. These results indicate that the polarities of the microenvironments around Trp and Tyr residues were not altered significantly by 2,4-TZD binding, but that the internal packing of HSA had changed.

#### 2.2.3. 3D fluorescence Analysis

**Figure 6:**
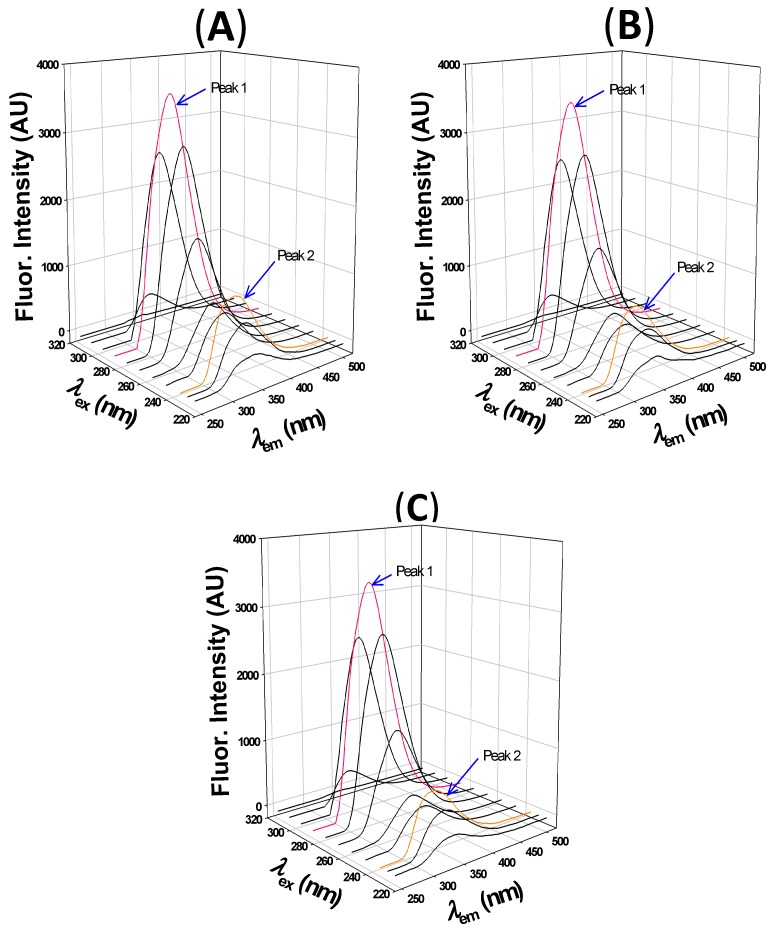
3D fluorescence spectra of HSA at different HSA to 2,4-TZD molar ratios. (**A**) HSA only, (**B**) molar ratio 1:5, and (**C**) molar ratio 1:10.

3D fluorescence is generally employed to monitor simultaneous changes in secondary (around peptide backbone) and tertiary (around aromatic amino acids) structures induced by ligand binding [48]. The 3D fluorescence spectra of HSA alone and at different HSA to 2,4-TZD molar ratios (1:5 and 1:10) are presented in Figure 6. Peak 1 indicates the 3D fluorescence spectral features of aromatic amino acid residues (Tyr and Trp), whereas peak 2 is a characteristic of the protein backbone. The results presented in Table 5 show that the fluorescence intensity of peak 1 was slightly quenched by 3.3% and 5.6% in the presence of 2,4-TZD at HSA to 2,4-TZD at molar ratios of 1:5 or 1:10 respectively. Moreover, the wavelength maximum of HSA was blue shifted by 3 nm in the presence of 2,4-TZD at both molar ratios, and the fluorescence intensity of peak 2 was quenched by 13.2% and 25.9% at HSA to 2,4-TZD molar ratios of 1:5 and 1:10 respectively. Furthermore, HSA fluorescence intensity peak was blue shifted by 3 nm at both molar ratios. We infer from these observations that the HSA structure had been altered by 2,4-TZD binding at the tertiary and secondary levels.

### 2.3. Effect of 2,4-TZD on the thermodynamic Stability and Unfolding of HSA

#### 2.3.1. Guanidinium Chloride (GdmCl)-induced Denaturation

**Figure 7:**
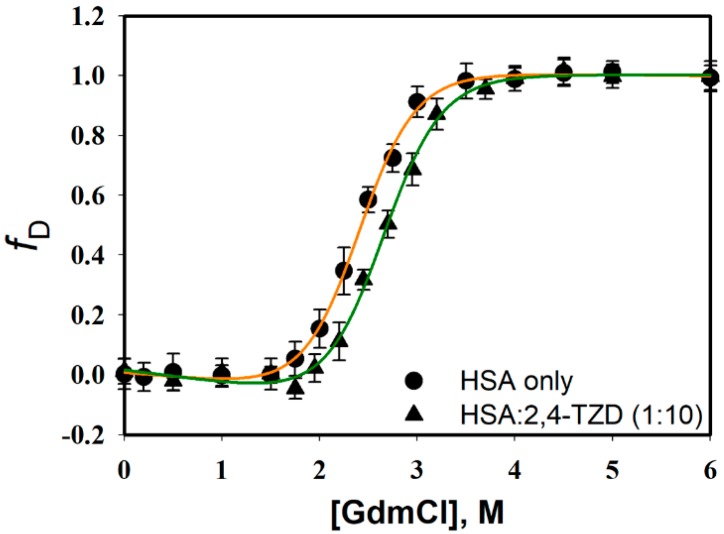
GdmCl-induced denaturation of HSA in the absence or presence of 2,4-TZD. *n* = 3.

Ligand to protein binding often causes changes in protein conformation and *thermodynamic* stability. To investigate the effect of 2,4-TZD binding on the thermodynamic stability of HSA, GdmCl-induced denaturation studies were performed in the presence or absence of 2,4-TZD; HSA at a fixed concentration was mixed with various concentrations of GdmCl and far-UV CD spectra were recorded. Raw CD data were used to extract the value of [*θ*]_222_ (a probe for the measurement of secondary structure); [*θ*]_222_ values were obtained for free HSA; and 2,4-TZD/HSA complexes were plotted as function of [GdmCl] (molar concentration of GdmCl). Figure 7 shows the normalized curve (plot of *f_D_* (the fraction of denatured protein molecule) versus [GdmCl]) for HSA and 2,4-TZD/HSA complex (1:1 ratio), obtained using the relation,

(8) $$f_{D}=\frac{y-y_{N}}{y_{D}-y_{N}}$$

where y is the observed optical property ([*θ*]_222_) at different [GdmCl], and *y_N_* and *y_D_* are native and denatured state optical properties, respectively.

GdmCl-induced transition curves were analyzed to obtained thermodynamic parameters, that is, Δ*G_D_*, *m*-values (dependence of Δ*G_D_* on GdmCl concentration), and *C_m_* (the denaturation midpoint) using a nonlinear least squares method (equation 12, in Materials and Methods). To analyze these curves, we assumed the following: (a) GdmCl-induced denaturation is a two-state process, i.e., Native ↔ Denatured, and (b) that Δ*G_D_* is linearly dependent on GdmCl. Values of the thermodynamic parameter obtained are detailed in Table 6. The thermodynamic data had good agreement with the published results for free HSA [49,50] and suggested the thermodynamic stability of HSA was slightly greater in the presence of 2,4-TZD.

#### 2.3.2. Analysis of Molecular Dynamics Simulation

**Figure 8:**
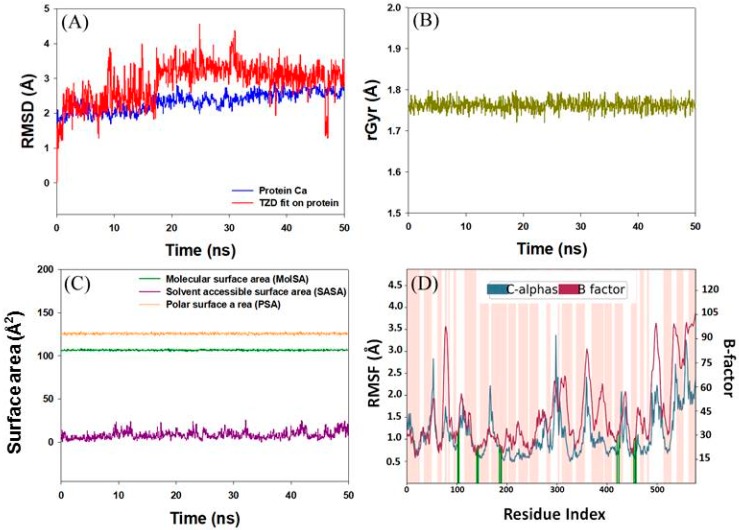
Molecular dynamics simulation of HSA-2,4-TZD complex. (**A**) Dependence of RMSDs of HSA in the absence and presence of 2,4-TZD on simulation time. (**B)** Fluctuation in radius of gyration (rGyr). (**C**) Variations in surface areas with respect to simulation time. (**D**) Dependence of RMSF, B-factor, and point of contact between HSA and 2,4-TZD as a function of simulation time.

The stability of the HSA-2,4-TZD complex was evaluated by performing molecular dynamics simulation for 50 ns at 300 K and atmospheric pressure (Figure 8). RMSD gives an insight into the structural conformation of a protein and indicates if the simulation has equilibrated or not. For a properly equilibrated and stable system, the fluctuations towards the end of the simulation should be within 1–3 Å for small globular proteins. Changes much larger than that, however, indicate that the protein is undergoing a large conformational change. In this study, we found that the RMSD value of HSA converges to a fixed value (~2.5 Å) towards the end of simulation, indicating a stable conformation of the protein (Figure 8A). Further, the analysis of ligand RMSD (fit to protein) indicates how stable the ligand with respect to protein and its binding pocket is. Here, we observed that the RMSD value of 2,4-TZD stabilized after initial fluctuations for 10 ns and was within the upper limit of 2 Å (Figure 8A). Further, the analysis of rGyr (radius of gyration) indicates that the radius of 2,4-TZD did not deviate significantly from the initial structure (~1.75 Å), implying that the overall compactness of the ligand remained unaltered during the course of simulation (Figure 8B). Moreover, the analysis of the molecular surface area (MolSA), polar surface area (PSA), and solvent accessible surface area (SASA) of HSA-2,4-TZD complex remain constant within the experimental limitations, thereby indicating the formation of a stable HSA-2,4-TZD complex (Figure 8C). Furthermore, the root means square fluctuation (RMSF) of HSA was evaluated to characterize the local changes along the protein chain (Figure 8D). The peaks in blue indicate the areas of that HSA that fluctuate the most during simulation. We observed all the major fluctuations which occurred in loop regions (indicated by the white bar), whereas the secondary structure of HSA (pink bars) was more rigid. It is significant to note that the RMSF of HSA corresponds with the experimentally determined X-ray crystal structure B-factor indicated by a red line. Furthermore, the vertical green lines on the *X*-axis of Figure 8D show the amino acid residues of HSA that interact with the 2,4-TZD. Overall, the molecular dynamics simulation studies indicate that HSA forms a stable complex with 2,4-TZD.

## 3. Materials and Methods

### 3.1. Materials

A lyophilized preparation of HSA (lot no A1887; fatty acid and globulin free) and 2,4-thiazolidinedione (2,4-TZD; 375004) were procured from Sigma Aldrich (St. Louis, MO, USA). Reagents of analytical grade were used to prepare buffers in deionized water.

### 3.2. Sample Preparation

Sodium phosphate buffer (20 mM, pH 7.4) was used to prepare HSA and 2,4-TZD stock solutions. Before preparing samples, HSA was dialyzed extensively in sodium phosphate buffer (20 mM) at 4 °C. An extinction coefficient ε280 = 36,500 M^−1^ cm^−1^ was used to determine protein concentrations [51].

### 3.3. Fluorescence Quenching Measurements

Jasco spectrofluorometer (FP-8300) equipped with a Peltier type temperature controller was used to measure HSA fluorescence quenching. Trp-214 of HSA was selectively excited at 295 nm and the spectra were recorded in the 300–450 nm range. Excitation and emission slits were set at 2.5 and 5 nm, respectively. HSA concentration was maintained at 4 μM during fluorescence measurements, but 2,4-TZD concentrations were varied from 0 to 56 µM. Measured fluorescence intensities were corrected for the inner filter effect as previously described [52]. All fluorescence quenching measurements were performed in triplicate.

### 3.4. Synchronous and 3-D Fluorescence Measurements

Synchronous and 3-D fluorescence studies were performed using a Jasco spectrofluorometer (FP-8300) as previously described [35]. To assess synchronous fluorescence of HSA (4 μM) in the presence or absence of 2,4-TZD (0–56 μM), spectra were recorded in the wavelength ranges of 260–340 nm and 280–400 nm. To investigate perturbation of Trp and Tyr residues, simultaneous excitation and emission wavelengths were recorded at Δλ (difference in wavelength) values of 15 and 60 nm, respectively. 3D fluorescence spectra of free-HSA (4 μM) and 2,4-TZD/HSA complex at molar ratios of 1:5 and 1:10 were obtained by recording emission spectra between 230–500 nm and an initial excitation of 220 nm with 5 nm increments. Synchronous and 3-D fluorescence measurements were repeated three times, however, for reasons of clarity, relevant portions of spectra are shown in Figure 6.

### 3.5. Far-UV Circular Dichroism Measurements

To investigate the influence of 2,4-TZD on the secondary structure of HSA, far-UV CD spectra were recorded in 1-mm path length cuvettes. Jasco J-815 spectropolarimeter (JASCO, Tokyo, Japan) pre-calibrated (with D-10-camphorsulphonic acid) was used to record CD spectra. To maintain a constant temperature of 25 °C throughout the experiment, we used a Peltier temperature controller. CD spectra were observed at HSA to 2,4-TZD molar ratios of 1:5 and 1:10 at a constant HSA concentration of 4 µM. The CD spectra were recorded over the range 200–250 nm and mean residual ellipticity (MRE) values were calculated using relation 9 [17],

(9) $${\left[\theta \right]}_{\lambda }=\frac{\theta_{\lambda }\;M_{o}}{10.c.l}$$

where, *θ_λ_* (in mdeg) is the observed ellipticity at wavelength λ, [*θ*]_*λ*_ (degcm^2^dmol^−1^) is the mean residual ellipticity, and *M_o_* is the mean residual weight (MRW) of protein. Protein concentration (mg/cm^3^) and path length (cm) are denoted by *c* and *l*, respectively. The percentages of the α-helix in HSA were calculated using;

(10) $$\%\alpha -helix=\left(\frac{-{\left[\theta \right]}_{222}-2340}{30300}\right)\times 100$$

where [*θ*]_222_ is the mean residual ellipticity at 222 nm.

### 3.6. Isothermal Titration Calorimetry (ITC) Measurements

A VP-ITC titration microcalorimeter (MicroCal, Inc. USA) was used obtain 2,4-TZD to HSA binding energies, as described by Rehman et al. [35]. Samples for ITC experiments were prepared by pre-dialyzing HSA in degassed 20 mM HEPES buffer (pH 7.4). 2,4-TZD was dissolved in the same buffer. For binding studies, 25 µM HSA and 1.25 mM of 2,4-TZD were used. Experimental data obtained were analyzed to determine the association constant (*K*a), entropy change (Δ*S^o^*), and enthalpy change (Δ*H^o^*). Three repetitions of the experiments were performed and the binding isotherms were fitted into the ‘one site’ binding model using MicroCal Origin 7.0 software to calculate the thermodynamic parameters.

### 3.7. Molecular Docking

The interaction between TZD and HSA was investigated by molecular docking using AutoDock4.2, as previously described [45]. The X-ray crystal of HSA (PDB Id: 1AO6; resolution 2.5 Å) was retrieved from the PDB database (https://www.rcsb.org/structure/1AO6). Before performing molecular docking, the protein structure of HSA was prepared by removing water and any other heterogeneous molecules. AutoDock tools were used to add essential hydrogen atoms, Kollman united atom type charges, and solvation parameters to the HSA molecule. A 75 × 60 × 80 Å along with *x*, *y* and *z* axis sized affinity grid map with a spacing platform 0.375 Å between grid points was created using the AutoGrid tool. The ligand (2,4-TZD) sdf file was downloaded from PubChem database (CID: 5437; available at https://pubchem.ncbi.nlm.nih.gov/), and after adding Gasteiger partial charges, merging non-polar hydrogen atoms, and defining rotatable bonds, it was converted to a pdbqt file. The energy of the ligand molecule was minimized by applying a universal force field. Molecular docking was performed using default values for AutoDock parameters. Lamarck Genetic Algorithm (LGA) and Solis and Wets protocols were used for local searching. A maximum of 2,500,000 energy calculations were performed per run and a total of 10 runs were computed. Population size and translational and quaternion/torsion steps were set at 150, 0.2 Å, and 5 respectively. All docking experiments were repeated in triplicate and mean error in analysis were reported in table. Figures of the docking analysis were prepared in Discovery Studio2.5 (BIOVIA, Wateridge Vista Drive, San Diego, CA, USA). Binding affinity (*K_b_*) was calculated from Gibbs free energy (Δ*G*) using the following relation.

(11) $$\Delta G=-RTlnK_{b}$$

### 3.8. Molecular Dynamics Simulation

Molecular dynamics simulation was performed using Desmond (Schrödinger, LLC, New York, NY, USA) for 50 ns using NTP ensemble at 300 K temperature and 1 bar atmospheric pressure as described earlier [53,54]. The docked HSA-2,4-TZD complex was considered as the initial conformation of the complex for the simulation. An orthorhombic simulation box was generated in the system builder in such a way that the boundaries of the box were at least 10 Å away from the protein. TIP3P explicit solvent model was employed to solvate the simulation box and proper counter-ions were added to neutralize the system. Further, 150 mM NaCl was added to the simulation box to mimic the physiological conditions. Before the start of simulation, the whole system was energy-minimized using OPLS3e forcefield till it converged to 1 kcal/mol/Å. The temperature and pressure were maintained constant using a Nose-Hoover Chain thermostat [55] and Martyna–Tobias–Klein barostat [56]. A time step was set at 2 fs and at every 10 ps, the energy and structure were recorded and saved in the trajectory. Three-dimensional structures and trajectories were analyzed using MAESTRO (Maestro, Schrödinger, LLC, New York, NY, USA).

### 3.9. GdmCl-induced Denaturation

For GdmCl-driven isothermal denaturation studies, HSA (10 μM) ellipticity in the absence or presence of 100 μM of 2,4-TZD was recorded at 222 nm and pH 7.4. Data were then used to calculate MRE using equation 9. The isothermal denaturation transition curves of free HSA and 2,4-TZD/HSA complex were reversible over the GdmCl concentration range examined. A non-linear least-squares method was then used to fit *y*(*g*) and [GdmCl] for thermodynamic parameters, i.e., Δ*G_D_*, *m_g_*, and *C_m_*, using the following relation [57].

(12) $$y(g)=\frac{y_{N}(g)+y_{D}(g)Exp^{\left[-\left(G_{D}+m_{g}[g]\right)/RT\right]}}{1+Exp^{\left[-\left(G_{D}+m_{g}[g]\right)/RT\right]}}$$

where *y*(*g*) is the observed HSA ellipticity at any GdmCl concentration, and *y_N_* and *y_D_* are the native and denatured state baselines of HSA obtained under the experimental conditions used to measure *y*(g).

In this equation, Δ*G_D_* is Gibb’s free energy change (when no denaturant was added), *m_g_* is the slope of the plot of δΔ*G_D_* vs. δ[g], *R* is the universal gas constant, and *T* is temperature in Kelvin. GdmCl-induced denaturations studies were made in triplicate. To analyze GdmCl-induced unfolding curves, two assumptions were made; (i) the native and denatured state baseline dependencies followed a linear model; (ii) protein unfolding was a two-state process (N↔D) [58,59].

## 4. Conclusions

In the present study, we systematically characterized the mechanism responsible for 2,4-TZD to HSA binding. Integrated spectroscopic, thermodynamic, and molecular docking studies were used to identify the 2,4-TZD binding site on HSA and to delineate the nature of the interaction involved in forming the 2,4-TZD/HSA complex. Our results show that 2,4-TZD quenches HSA fluorescence by forming a ground-state HSA-2,4-TZD stoichiometric complex of molar ratio 1:1. Reductions in quenching (*K_SV_*) and binding constants (*K_b_*) on increasing temperature showed that 2,4-TZD/HSA complex formation results in static quenching. Thermodynamic parameters obtained from fluorescence quenching experiments at different temperatures correlated well with calorimetric results and showed that 2,4-TZD/HSA complex formation is spontaneous and driven by an entropy change. 2,4-TZD displayed moderate binding affinity of 1.69 × 10^3^ M^−1^ and a Gibbs free energy change of −4.4 kcal mol^−1^. Molecular docking analysis showed that 2,4-TZD binds at subdomain IB of HSA by hydrogen bonding and van der Waal’s interactions. FRET analysis confirmed 2,4-TZD binds near Trp214, which predicts quenching. Furthermore, a blue shift observed in the 3-D fluorescence spectrum of HSA and an increase in circular dichroism molar ellipticity confirmed 2,4-TZD binding induced a conformational change in HSA; and an increase in *C_m_* value from GdmCl-induced denaturation of HSA in the presence of 2,4-TZD indicated binding increased the thermodynamic stability of HSA. Overall, our results show that 2,4-TZD binds efficiently to HSA and predicts that 2,4-TZD is likely to be transported to different parts of the body by HSA in plasma. We also observed that the O-atoms and –NH group of 2,4-TZD have played crucial roles in interacting with HSA. Thus, any substitution of 2,4-TZD should be performed in such a way as to avoid these positions and maximize the interaction between HSA and TZD. Furthermore, 2,4-TZD induced conformational change in HSA which probably influences the binding of other molecules. We believe the findings of this study provide potential insight into the mechanism responsible for the binding of 2,4-TZD to HSA and improves the understating of its effect during transport and distribution in the blood. Additionally, our finding on the binding affinity of 2,4 TZD may provide valuable information about dosage forms necessary to achieve desire response and to reduced toxic side effects.

## Figures and Tables

**Table 1:** Binding and thermodynamic parameters of 2,4-TZD/HSA binding as determined by fluorescence quenching. Values are means of triplicates ± SD.

Parameter	298 K	303 K	310 K	315 K
*K_SV_* × 10^3^ (M^−1^)	2.67 ± 0.21	2.10 ± 0.18	1.63 ± 0.14	1.27 ± 0.10
*k_q_* × 10^11^ (M^−1^ s^−1^)	4.67 ± 0.39	3.68 ± 0.33	2.85 ± 0.27	2.22 ± 0.21
*K_b_* × 10^3^ (M^−1^)	1.69 ± 0.15	2.75 ± 0.21	3.90 ± 0.26	8.42 ± 0.29
*n* (binding stoichiometry)	0.950 ± 0.02	1.02 ± 0.03	1.08 ± 0.02	1.18 ± 0.04
Δ*H^o^* (kcal mol^−1^)	16.34 ± 0.96			
*T*Δ*S^o^* (kcal mol^−1^)	20.73 ± 1.32	21.07 ± 1.08	21.56 ± 1.27	21.91 ± 1.18
Δ*G^o^* (kcal mol^−1^)	−4.39 ± 0.63	−4.73 ± 0.44	−5.22 ± 0.59	−5.57 ± 0.52

**Table 2:** Binding and thermodynamic parameters obtained by isothermal calorimetric titration of 2,4-TZD with HSA at 298 K. Values are means of triplicates ± SD.

*K_a_* (M^−1^)	Δ*H^o^*, (kcal mol^−1^)	*T*Δ*S^o^* (kcal mol^−1^)	Δ*G^o^* (kcal mol^−1^)
2.1 ± 0.11 × 10^3^	−14.4 ± 1.21	−8.65 ± 0.85	−5.75 ± 0.48

**Table 3:** FRET parameters for the interaction between 2,4-TZD and HSA.

*J* (M^−1^ cm^3^)	*R_o_* (nm)	*r* (nm)
3.08 × 10^−14^	2.96	4.08

**Table 4:** Molecular docking between HSA and 2,4-TZD using AutoDock4.2.

Nature of Interaction	Category of Interaction	Distance (Å)	Δ*G* (kcal mol^−1^)	*K_b_* × 10^3^ (M^−1^)
Tyr148:HN-Lig:O	Hydrogen Bond	2.07 ± 0.06	−4.61 ± 0.54	1.69 ± 0.15
Lig:H-Ser193:O	Hydrogen Bond	2.57 ± 0.08		
Ala194:CA-Lig:O	Carbon Hydrogen Bond	3.38 ± 0.08		

**Table 5:** 3D fluorescence parameters for the interaction between HSA and 2,4-TZD.

	Peak 1			Peak 2		
	Peak Position (λex/λem) nm/nm	Fluorescence Intensity (AU)	Stokes Shift (nm)	Peak Position (λex/λem) nm/nm	Fluorescence Intensity (AU)	Stokes Shift (nm)
HSA	280/335	3607	55	230/327	1077	97
HSA:2,4-TZD (1:5)	280/332	3488	52	230/324	935	94
HSA:2,4-TZD (1:10)	280/332	3406	52	230/324	809	94

**Table 6:** Thermodynamic parameters associated with the GdmCl-induced denaturation of HSA in the presence or absence of 2,4-TZD at pH 7.4 and 25 °C. Values are means of triplicates ± SD.

	Δ*G_D_^o^*, kcal mol^−1^	*m_g_*, kcal mol^−1^ M^−1^	*C_m_*, M
HSA only	4.94 ± 0.32	2.05 ± 0.14	2.40 ± 0.12
HSA:2,4-TZD (1:1)	5.30 ± 0.48	2.01 ± 0.17	2.61 ± 0.19

## Abbreviations

HSA	Human Serum Albumin
2,4-TZD	2,4-thiazolidinedione
ITC	Isothermal Calorimetry
CD	Circular Dichroism
FRET	Förster Resonance Energy Transfer
LGA	Lamarck Genetic Algorithm
